# Experimental Demonstration of Low-Uncertainty Calibration Methods for Bragg Grating Interrogators

**DOI:** 10.3390/s18061895

**Published:** 2018-06-10

**Authors:** José Luis de Miguel, Juan Galindo-Santos, Concepción Pulido de Torres, Pedro Salgado, Aitor V. Velasco, Pedro Corredera

**Affiliations:** 1 Instituto de Óptica, CSIC, C/Serrano 121, 28006 Madrid, Spain; j.galindo@csic.es (J.G.-S.); a.villafranca@csic.es (A.V.V.); p.corredera@csic.es (P.C.); 2 Instituto de Estructura de la Materia, CSIC, Serrano 123, 28006 Madrid, Spain; conchi.pulido@io.cfmac.csic.es; 3 Instituto Mexicano del Petróleo—IMP, Ciudad de México 07730, México; psalgado@imp.mx

**Keywords:** fiber-optic sensors, fiber Bragg gratings interrogators, absolute calibration

## Abstract

In this paper we propose and demonstrate two alternative methods for the high-precision calibration of fiber Bragg grating (FBG) interrogators. The first method is based on the direct comparison between the wavelength measurements of the interrogator under test and a calibrated wavemeter, while analyzing a simulated symmetric Bragg grating constructed by a tunable filter and a fiber mirror. This first method is applicable to most commercial systems but presents an uncertainty limited by the spectral width and the wavelength stability of the tunable filter. The second method consists in measuring multiple reference absorption lines of calibrated absorption gas cells. This second method presents lower uncertainties, limited only by the optical resolution of the interrogator and the wavelength uncertainty of the reference cell absorption lines. However, it imposes more restrictive requirements on the interrogator software. Both methods were experimentally demonstrated by calibrating multiple commercial systems, reaching uncertainties down to 0.63 pm at a central wavelength of 1550 nm.

## 1. Introduction

Fiber-optic sensors based on fiber Bragg gratings (FBG) are nowadays ubiquitous as quasi-distributed strain and temperature sensors, as they outperform traditional technologies in terms of mechanical advantages, multiplexing capabilities, and remote operation [1,2]. An FBG is made by inscribing a periodic modulation of the effective refractive index in the optical fiber core, and operates in reflection as a narrow band-pass filter. The central wavelength of the reflected band-pass, also known as the Bragg wavelength, depends on the pitch of the grating and the effective refractive index. Any change in the index or grating pitch caused by external effects, such as strain or temperature, results in a Bragg wavelength shift, making FBG very attractive for sensing applications. In particular, FBG applications range from in situ sensors in the medical industry for monitoring biological functions, tumor detection and treatment, and post-trauma care, through to structural health monitoring in aerospace and ship structures, civil and mechanical engineering, and geodynamical monitoring [3,4,5,6,7,8,9].

Throughout the last decade, a large variety of FBG interrogators, aimed at recovering the wavelength-encoded information from one or several multiplexed FBG sensors, have been developed [2,10]. Currently, two main interrogator technologies have been consolidated in commercial equipment. The first setup is based on a spectrometer which analyzes the response of the FBG to a broadband source, whereas the second approach is based on a tunable laser and a synchronized photodetector. In general terms, the first setup provides lower wavelength resolution, but has a good long-term stability. Oppositely, the second setup provides better resolution, a wider wavelength window, and a longer distance range (due to the higher source power), thus allowing for a larger number of sensors to be interrogated simultaneously. However, it has an inferior long-term wavelength stability. This drawback has been overcome by the incorporation of one or various internal self-referencing devices, such as acetylene gas cells and ultrastabilized Bragg gratings [2].

In 2016, the International Electrotechnical Commission (IEC) adopted a new standard, IEC 61757-1-1:2016 [11], that specifies the most relevant features and characteristics of an FBG-based strain sensor and defines the procedures for their determination. Furthermore, the following key performance parameters of the corresponding FBG interrogators are identified in the document: basic operating principle, method of peak determination, stability of the Bragg wavelength measurement, repeatability of the Bragg wavelength measurement, range of FBG peak detection, sampling rate, maximum number of sensors per channel/per instrument, Bragg peak shift resolution, dynamic range, available optical power budget, minimum detectable FBG signal-to-noise ratio, number of channels, availability of the characteristic FBG spectrum, and required/suggested calibration interval. For reference, Table 1 summarizes a selection of these parameters (those more relevant to the present study) in state-of-the-art commercial systems, as obtained from the specifications provided by the manufacturers.

In order to assess the performance of an FBG interrogator, it needs to undergo a calibration process traceable to a primary standard, determining the repeatability and the reproducibility of the measurements as well as any systematic error the system may contain. Once measured, this bias can be used as a correction offset [12,13,14]. Wavelength calibration is also of paramount importance in order to cope with possible drifts, that is, deviations of the initial reference value of static or quasi-static measurements influenced by drifts in the FBG interrogator and sensor [11]. Careful calibration of the interrogator is essential since a wavelength measurement uncertainty as small as 1 pm may typically lead to an uncertainty of nearly 1 microstrain or 0.1 °C [2]. Traditionally, FBG interrogator calibration is performed by a straightforward combination of previously-calibrated FBG gratings and optical spectrum analyzers (OSAs), but this approach provides limited accuracy [15]. In particular, properly-calibrated OSAs typically limit uncertainty to 10 pm, whereas OSAs which are not accurately calibrated to a known wavelength reference increase measurement uncertainty up to 1 nm. Furthermore, previously-calibrated Bragg gratings are subject to deviations from their nominal values due to environmental fluctuations that result in Bragg wavelength drifts and changes in their spectra. Methods based on lasers referenced to frequency combs have also been proposed [16], although they are only applicable to FBG interrogators incorporating an internal spectrometer, and they require complex and expensive instrumentation. Finally, some manufacturers calibrate their interrogation units by using hydrogen cyanide H^13^C^14^N gas absorption cells [17], but this calibration is limited to wavelengths within the C band of telecommunications (1530 nm to 1565 nm).

In this work, we aim to overcome these limitations by presenting two low-uncertainty methods for the calibration of FBG sensor interrogators. The first method is based on the direct comparison between the wavelength measurements of the interrogator under test and a calibrated wavemeter, while analyzing a simulated symmetric Bragg grating composed of a tunable filter and a fiber mirror. This method is applicable to most commercial systems, although it requires a high resolution calibrated wavemeter traceable to a primary standard. The second method consists in measuring multiple reference absorption lines of calibrated absorption gas cells covering the whole wavelength range of the interrogator. The second method provides a lower uncertainty but requires that the commercial system is equipped with software post-processing tools capable of characterizing absorption lines or that new software code is specifically written to access this information, hence hindering the calibration of instruments lacking these tools. Both are experimentally demonstrated by calibrating multiple commercial systems.

## 2. Materials and Methods

### 2.1. First Method: Simulated Tunable Bragg Grating

**Figure 1:**
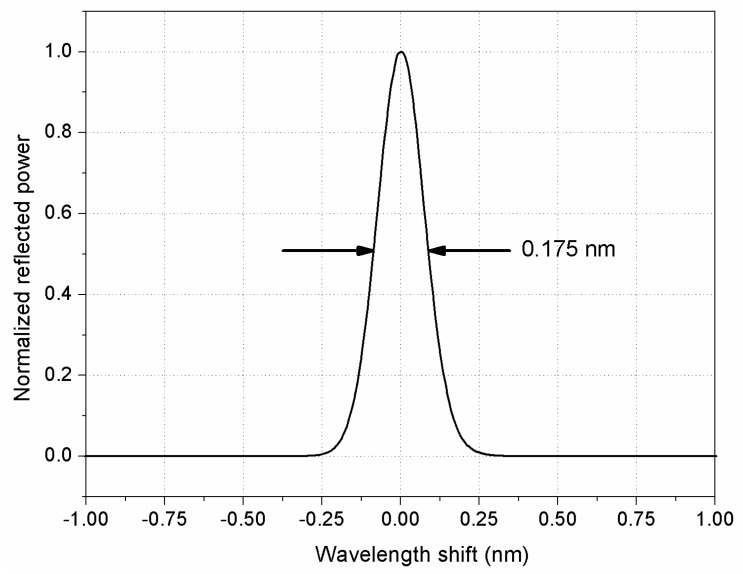
Spectrum and band-width of the simulated fiber Bragg grating (FBG) formed by the tunable filter and the fiber mirror.

The first of the two implemented methods described herein is based on simulating a tunable fiber Bragg grating by using a tunable filter and a fiber mirror. This ensemble behaves analogously to an actual fiber Bragg grating by reflecting particular wavelengths of light with comparable bandwidths [18], while presenting the additional advantage of being fully tunable and providing a symmetrical spectrum. The use of the mirror is a requirement for the type of interrogators used in this study, which operate in reflection and not in transmission, but it also adds the benefit of a narrower reflected signal as a consequence of the double pass through the filter. Figure 1 shows the shape and spectral width of the simulated FBG formed by the tunable filter and the fiber mirror used in our setup, as detailed below. A full-width at half-maximum (FWHM) bandwidth of 0.175 nm is achieved, similar to most common Bragg gratings used by the interrogators under calibration. The combination of the tunable filter and the fiber mirror results in a symmetric spectrum, typically Gaussian or Lorentzian. The slope symmetry of the ascending and descending flanks facilitates accurate peak measurement, regardless of the particular methodology implemented by the interrogator [10,15].

**Figure 2:**
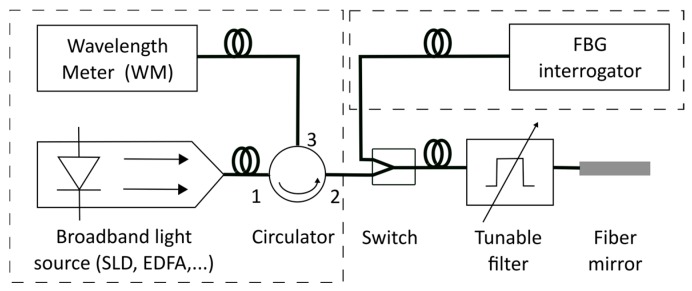
Setup of the first calibration method, based on a simulated fiber Bragg grating.

The simulated FBG is alternately fed with the signal produced by a broadband source, such as a superluminescent diode (SLD) or the spontaneous emission from an Erbium Doped Fiber Amplifier (EDFA), and with the signal produced by the FBG interrogator under test. The switch further prevents any cross-talk between the broadband light source and the optical source of the FBG interrogator under test. In the first case (i.e., with SLD or EDFA illumination), the reflected peak is measured with a calibrated wavelength meter (WM), which acts as the reference to which the interrogator is compared. Furthermore, by performing both measurements under the same conditions, any environmental effects which may affect the simulated Bragg grating are inherently incorporated into both measurements and do not result in deviations of the correction constants. The setup is depicted in Figure 2.

The wavelength obtained with the FBG interrogator under test ($\lambda_{FBGI}$) and the wavelength measured with the WM ($\lambda_{WM}$) are compared for multiple wavelengths, selected by the tunable filter along the range of measurement of the FBG interrogator. The correction constant of the FBG interrogator at any wavelength ($K_{\lambda }$) is hence calculated as:

(1) $$K_{\lambda }=\lambda_{FBGI}-\lambda_{WM}$$

The identified components of uncertainty in the determination of $K_{\lambda }$ are the WM calibration, the WM accuracy due to the linewidth of the simulated tunable FBG, and the repeatability of the reference, all of them affecting $\lambda_{WM}$, as well as the optical resolution, the display resolution, and the on-off repeatability of the interrogator affecting $\lambda_{FBGI}$. The main sources of uncertainty arise as a consequence of the spectral width of the simulated tunable FBG, which affects the WM accuracy and the repeatability of the reference. For the particular case of a WM based on a Michelson interferometer and a He-Ne reference laser [19,20,21], the attainable wavelength accuracy is influenced by several sources of systematic errors [22]: the uncertainty in the knowledge of the reference laser wavelength, the accuracy of measuring the refractive index of air, the alignment of the reference laser beam and the input laser beam in the Michelson interferometer, diffraction effects along the beam path, and the fringe counting error, which is inversely proportional to the signal bandwidth.

Compared to using calibrated FBGs as wavelength references [15], our method presents the advantage of not being affected by strain and temperature, as the wavelength measured by the interrogator is compared to a reference provided by the wavemeter in the same environmental conditions, instead of a nominal value which may not represent the actual operating conditions of the calibrated FBGs. Although, in principle, a set of athermal calibrated FBGs could be used instead of the proposed simulated FBG, the latter presents the advantage that its peak wavelength can be finely or coarsely tuned to any value within the operating range of the interrogator, and that the shape of the spectrum is symmetrical (see Figure 1) and kept constant throughout the whole range. This means that the component of uncertainty in the determination of $\lambda_{WM}$ due to the FBG linewidth, which is one of the main components of uncertainty in the determination of $K_{\lambda }$, is constant, and thus there is no need to characterize the reflection spectra of all of the FBGs to ensure that their spectral characteristics remain unaltered.

### 2.2. Second Method: Gas Absorption Cell

**Figure 3:**
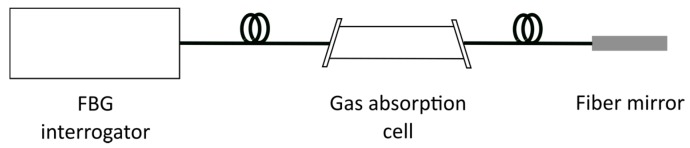
Setup of the second calibration method, based on calibrated absorption gas cells.

The second method is based on the direct measurement with the FBG interrogator under test of multiples absorption lines of molecular gas cells operating around 1550 nm, as shown by the setup depicted in Figure 3. Molecular gas cells inherently provide low-uncertainty wavelength references, as their spectral features are intrinsically defined by the vibrational-rotational characteristics of the gas species [23,24,25,26]. Although this approach is well known, in our case we used a plurality of gas cells which cover the whole operating range of modern commercial interrogators, as opposed to the limited C-band coverage of gas cells traditionally used.

The correction constant of the FBG interrogator at any wavelength ($K_{\lambda }$) is calculated as the difference between the FBG interrogator measurement ($\lambda_{FBGI}$) and the cell nominal values ($\lambda_{REF}$):

(2) $$K_{\lambda }=\lambda_{FBGI}-\lambda_{REF}$$

**Figure 4:**
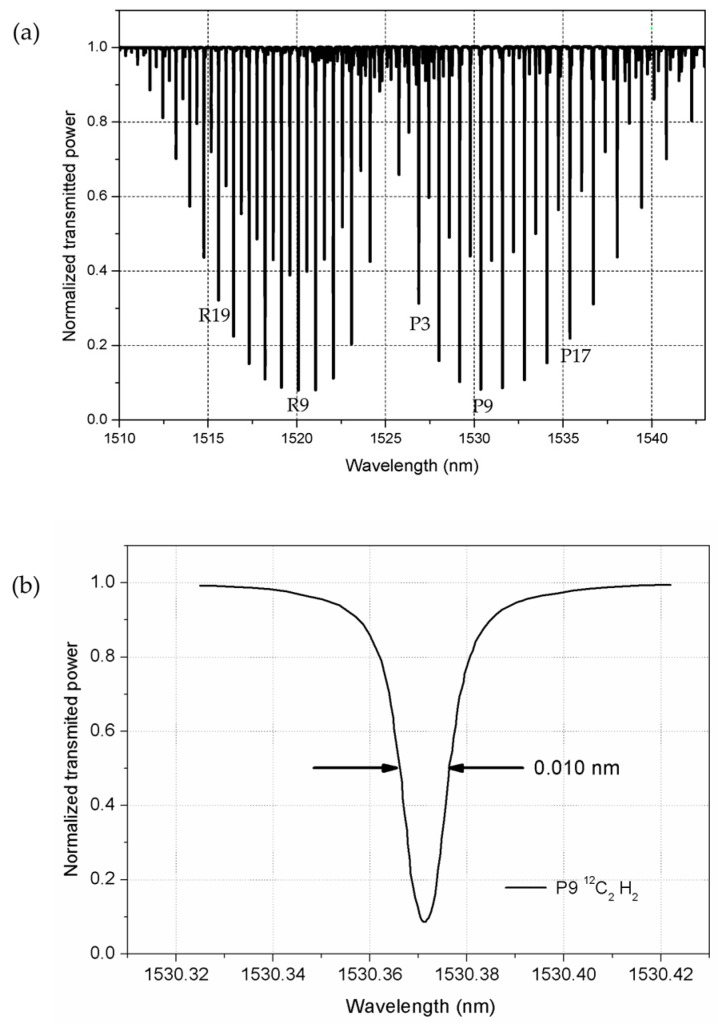
(**a**) Acetylene absorption spectrum; and (**b**) the detailed spectrum of its line P9.

For illustrative purposes, Table 2 includes some of the molecular gas cells most frequently used as reference material in the C and L bands for telecommunications. A typical acetylene absorption spectrum (^12^C_2_H_2_) is shown in Figure 4a, covering the 1510 nm–1540 nm range. The subset of absorption lines used in the particular experiments presented in this work is also indicated in Figure 4a (namely, lines R19, R9, P3, P6, and P17). Figure 4b shows in greater detail one of the selected absorption lines, centered at 1530.3711 nm, with an FWHM bandwidth of 0.01 nm. They were all obtained with a tunable laser and a calibrated wavemeter.

The uncertainty in the determination of the correction constant is mainly affected by the optical resolution of the FBG interrogator and the wavelength uncertainties of the reference absorption lines. Therefore, this second method provides lower uncertainties than the first, but it requires that the software post-processing tools integrated in the FBG interrogator be capable of characterizing absorption lines. Gas absorption cells also present a lower sensitivity to environmental variations than calibrated Bragg gratings traditionally used for interrogator calibration. The pressure-induced shift and broadening of the different lines of the gases used was extensively studied by Swann and Gilbert [26,27,28]. Calibrated gas absorption cells designed for the wavelength calibration of instruments use fixed internal pressure to match the bandwidth of the reference to the instrument resolution. Moderate thermal changes can slightly modify the pressure-induced shift and broadening of the molecular line. The temperature dependence of the pressure-induced shift, ∆λ(*T*), is [26]:

(3) $$\Delta \lambda (T)=\Delta \lambda (Tm)\sqrt{T/Tm}$$

where ∆λ(*T_m_*) is the pressure-induced shift measured at temperature *T*_m_ and the temperatures *T* and *T_m_* are in Kelvin. From this equation, it can be seen that the line center is fairly insensitive to temperature changes. A change of 50 K around the typical calibration conditions (23 ± 2 °C) results in a ±0.3% change in the pressure shift in the case of acetylene, which is negligible compared with other sources of uncertainty [27]. In the case of CO, this temperature change would cause an 8% change in the pressure-induced shift, which corresponds to a maximum wavelength change of 0.2 pm. For the standard calibration temperature conditions of 23 ± 2 °C, this means a 0.04 pm change.

## 3. Results

### 3.1. Devices Under Test

The two methods described above were applied to calibrate several commercial systems: two static interrogators (sm125-500) and one dynamic interrogator (sm130-700) manufactured by Micron Optics, as well as a static portable equipment (FS42) manufactured by HBM. The static sm125-500 features a swept-wavelength laser [29], which scans the 1510 nm–1590 nm wavelength range with a frequency of up to 2 Hz. The optical resolution of the system is 0.8 pm and the display resolution is 0.01 pm. The interrogator supports continuous on-board NIST (National Institute of Standards and Technology) traceable wavelength reference components, including an acetylene gas cell (^12^C_2_H_2_) and a Fabry-Perot. All units are externally calibrated after manufacture following a standard test set that makes use of hydrogen cyanide (HCN) gas cells. The peak or valley values are determined as the central wavelength of the spectral feature that exceeds a specified threshold value (typically, 3 dB from the maximum amplitude). The dynamic sm130-700 is in many respects similar to the static interrogator described above, but its swept-wavelength laser scans the 1510 nm–1590 nm wavelength range with a frequency of up to 1000 Hz. Besides, it is not equipped with a gas cell as a reference. The optical resolution of the system is 0.8 pm and the display resolution is 0.01 pm. The portable FS42 also employs continuous swept laser scanning technology and a NIST traceable wavelength reference gas cell (HCN). It scans the 1500 nm–1600 nm range at a frequency of 1 Hz. The optical resolution of the system is 1 pm and the display resolution is 0.01 pm.

### 3.2. First Method: Simulated Bragg Grating

In our setup, we used a JDSU (San Jose, CA, USA) Model MAPF+1GGP01FA tunable filter and a FORF-31P-1300/1550-9/125-s-3a-1-1 (OZ Optics, Ottawa, ON, Canada) gold tipped fiber total reflector to simulate the Bragg grating. The spectral width (FWHM) of the tunable filter alone is of the order of 0.25 nm, but the convolution with the signal reflected by the fiber mirror narrows it to about 0.175 nm, similar to the spectral width of typical FBGs. The resulting simulated FBG was tuned in approximately 5-nm steps in the wavelength range from 1510 nm–1590 nm. As a broadband source, we used a Thorlabs (Newton, NJ, USA) 14 Pin Butterfly Packaged SLD for the 1510 nm–1570 nm range and an Accelink Technologies (Wuhan, China) Model EFDA-BA-L-25-18-FC/APC Erbium doped fiber amplifier for the 1570 nm–1590 nm range. The reflected signal was measured with an EXFO (Quebec, QC, Canada) WA-1650 wavemeter, previously calibrated in-house using a self-referenced optical frequency comb [16]. The switch used was a JDS Fitel (San Jose, CA, USA), with a 10^−4^ output power asymmetry and a flat spectral response. The uncertainty budget, calculated according to the ISO/BIPM guidelines [14], is summarized in Table 3. The repeatability of the reference was obtained by experimentally estimating the lower and upper bounds of the wavelength of the reference, the simulated tunable FBG, as measured with the wavemeter, and assuming a uniform or rectangular distribution of possible values. The on-off repeatability of the interrogator was estimated in a similar way.

### 3.3. Second Method: Gas Absorption Cell

In our setup, we used two gas absorption cells, namely an acetylene ^12^C_2_H_2_ cell (NIST Standard Reference Material 2517a) for the 1515 nm–1535 nm region [22] and a fiber coupled three-gas cell, H^12^C^14^N + ^12^C^16^O + ^13^C^16^O (pressures of 5 Torr, 150 Torr and 150 Torr, respectively), manufactured by Wavelength References with NIST traceability, for the 1535 nm–1570 nm region. A series of reference absorption lines were measured repeatedly with the FBG interrogator ($\lambda_{FBGI}$) for periods of time ranging between 5 min and 2 h. No significant differences were observed as a function of the measuring time, proving a very high measurement stability. This can be seen in Table 4, which contains the uncertainty budget [14] at $\lambda_{WM}$ ≈ 1530 nm. $\lambda_{REF}$ was obtained from the data tabulated by NIST [23]. Values corresponding to the ^12^C^16^O absorption cell were corrected for pressure-induced shift [26] and the uncertainty ascribed to the pressure-shift coefficient was quadratically added to the uncertainty of the absorption line value.

## 4. Discussion

**Figure 5:**
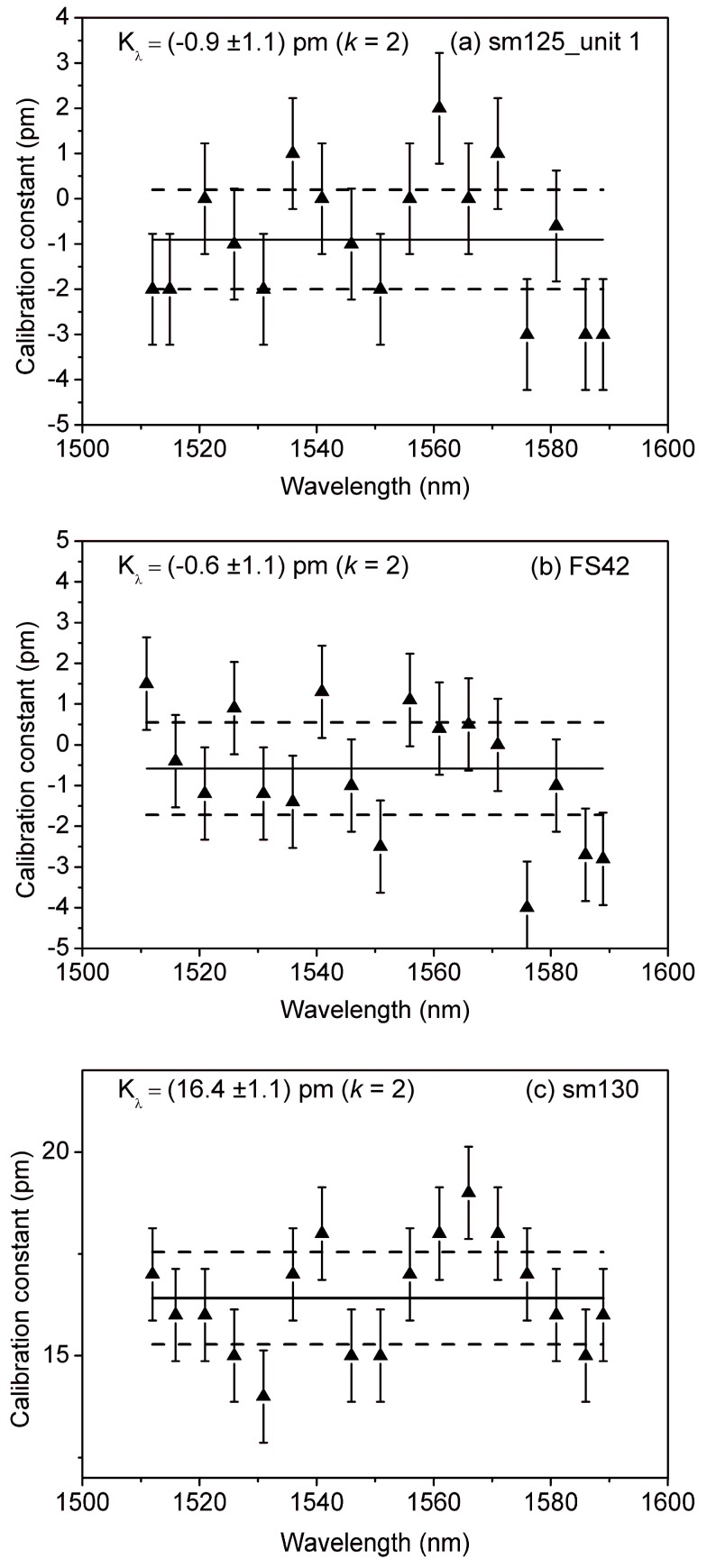
Correction constants with wavelength-dependent uncertainties for the devices under test, as determined by the first calibration method: (**a**) sm125-500; (**b**) FS42; (**c**) sm130-700. The mean correction constant (solid line) and expanded uncertainty (*k* = 2, dashed line) are also depicted.

**Figure 6:**
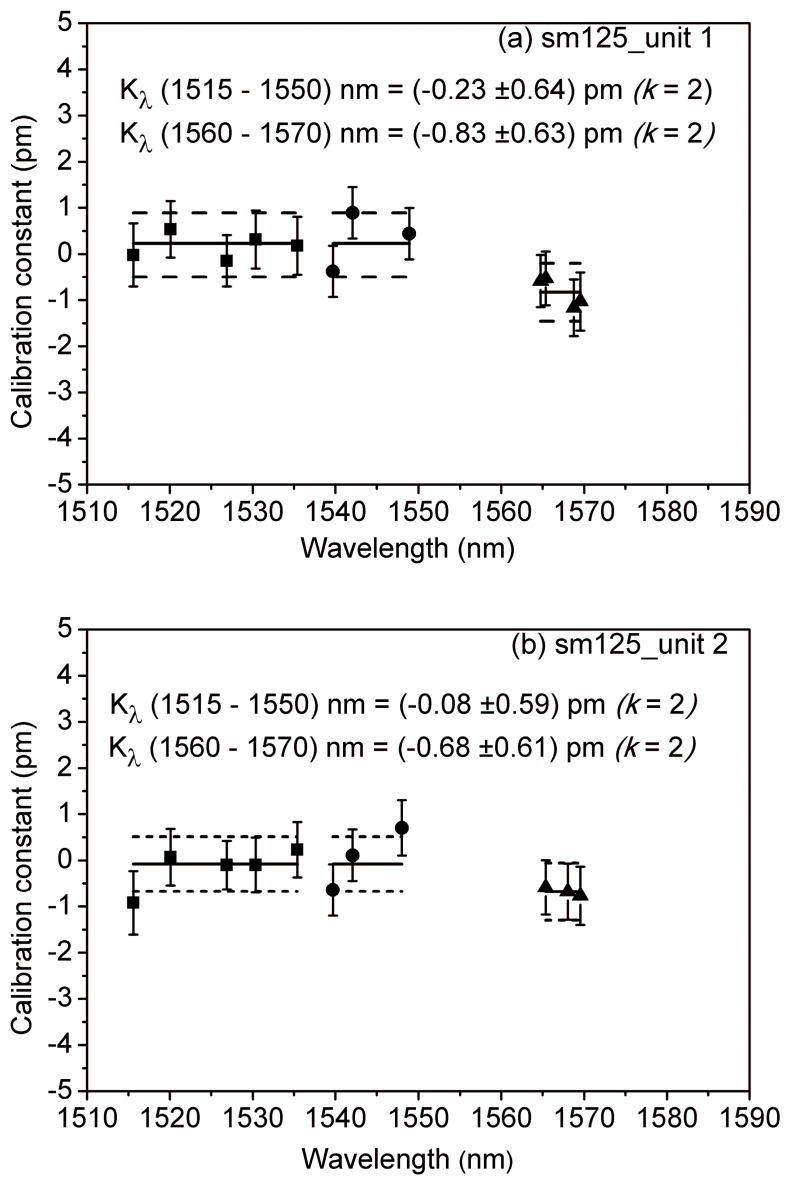
Correction constants with wavelength-dependent uncertainties for the devices under test, as determined by the second calibration method, using acetylene (squares), hydrogen cyanide (circles), and carbon monoxide ^12^C^16^O (triangles) gas absorption cells: (**a**) sm125-500 unit 1; (**b**) sm125-500 unit 2. The mean correction constants (solid line) and expanded uncertainties (*k* = 2, dashed line) are also depicted.

The experimental demonstration of both calibration methods is presented in Figure 5 and Figure 6, respectively. Figure 5 shows the results of calibrating two of the static interrogators, each one produced by a different manufacturer, and the dynamic interrogator, using the simulated Bragg grating. The correction constants with wavelength-dependent uncertainties, as well as the mean correction constant (solid line) and expanded uncertainty (*k* = 2, dashed line), are depicted for each of the devices under test.

The mean correction constant for each of the two static interrogators (i.e., sm125 and FS42) are below 1 picometer and its value lies within the expanded uncertainty (*k* = 2), unlike that of the dynamic interrogator (i.e., sm300), which is 16.4 pm. This difference reflects that only the two static interrogators are equipped with an on-board traceable wavelength reference that provides continuous calibration.

Figure 6 shows the results of calibrating two units of the same static interrogator model using gas absorption cells. It is worth mentioning that they are the only ones, among the four calibrated in this study, equipped with software post-processing tools capable of characterizing absorption lines, which is a prerequisite for the application of this method. The correction constants with wavelength-dependent uncertainties, as well as the mean correction constant (solid line) and expanded uncertainty (*k* = 2, dashed line), are depicted for each of the devices under test.

The accuracy of this method enables the determination of range-dependent correction constants, one for the 1515 nm–1550 nm range and another for the 1560 nm–1570 nm range. For both units, the correction constant in the 1560 nm–1570 nm range is larger and its value lies outside the expanded uncertainty. This may be attributed to the design of the commercial interrogator, which incorporates an internal acetylene (^12^C_2_H_2_) gas cell for self-calibration in the 1510 nm–1540 nm range, and undergoes an in-factory external calibration with HCN gas cells. As a consequence, the interrogator response in the previously-calibrated wavelength ranges is accurately characterized, leading to small correction constants (0.08 pm and 0.23 pm, respectively), whereas any measurements outside these ranges require a greater correction (0.83 pm and 0.69 pm, respectively). In measurements with very high accuracy requirements, such as sea temperature vertical profiling [30], incorporating these corrections may have a significant impact. Furthermore, this correction could be expected to grow as the wavelength window of the FBG interrogator grows outside the wavelength range where the internal reference cell works.

The mean correction constants and expanded uncertainties (*k* = 2) for the four commercial systems are summarized in Table 5. While the second method provides lower uncertainties, the fact that it requires that the FBG interrogators be capable of handling absorption lines limits its applicability. Instead, the use of the simulated Bragg grating is applicable to most types of commercial FBG interrogators and presents an advantage over previously used calibration methods, namely using calibrated FBGs, of being insensitive to strain and temperature changes and independent of the implemented technology for peak determination.

## 5. Conclusions

In this paper, we proposed two alternative low-uncertainty methods for the calibration of fiber Bragg grating (FBG) sensor interrogators and experimentally demonstrated their application in the calibration of four commercial systems. The first method is based on the direct comparison between the wavelength measurements of the interrogator under test and a calibrated wavemeter, while analyzing a simulated tunable symmetric Bragg grating composed of a tunable filter and a fiber mirror. This first method presents an uncertainty of 1.1 pm, limited by the spectral width of the tunable filter and the wavelength stability of filter and wavemeter. The second method consists in measuring multiple reference absorption lines of calibrated absorption gas cells covering the operating wavelength range of modern commercial FBG interrogators. This second method presents an even better uncertainty (down to 0.6 pm), providing a more sensitive calibration of high-accuracy Bragg grating interrogators and enabling wavelength-dependent correction constants. However, despite providing a more accurate characterization of the devices, the second method is only applicable to commercial systems equipped with software post-processing tools that are capable of characterizing absorption lines, or requires that new software code be written to access this information, which is impractical for most calibration laboratories. The first method is applicable to most commercial systems.

The calibration of four commercial FBG interrogators shows the importance of having an on-board wavelength reference cell for self-calibration. The application of the first method to two interrogators equipped with such a wavelength reference resulted in correction constants below 1 picometer and with values that lie within the expanded uncertainty, while the correction constant of the interrogator without it yielded a calibration constant above 16 pm. The highest accuracy of the second method allowed for establishing different correction constants for different wavelength ranges. This is attributable to a different behavior of the interrogators outside the range covered by the internal self-calibration gas cell. Introducing these corrections is relevant in measurements with very high accuracy requirements or in interrogators operating at wavelengths far from where the internal reference cell works.

These results show remarkable potential for providing much-needed traceability in diverse applications such as structural health monitoring, as well as in the aerospace, oil and gas, transport, and civil engineering sectors.

## Figures and Tables

**Table 1:** Specifications for some commercially available interrogators in the 1550-nm region.

Parameter	Micron Optics sm125-500	Micron Optics sm130-700	Micron Optics si155 Standard	HBM FS22—Industrial BraggMETER (Static)	HBM FS22—Industrial BraggMETER	HBM FS42—Portable BraggMETER	Smart Fibers SmartScope FBG Interrogator	Smart Fibers SmartScan FBG Interrogator	FAZT I4G Interrogator	Optilab FSI-RM-18	BaySpec WaveCapture^®^ Turn-key FBG Sensing	Ibsen I-MON 256 (512) OEM Monitors
Operating principle	Swept laser										Spectrometer	
Stability (*) (Reproducibility) (pm)	±1	±2 typically, ±5 max	±1	±1	±5	±2.5	3	<5	<±1	±2.5		
Repeatability (pm)	±0.5 at 1 Hz; ±0.2 at 0.1 Hz	±1	±1	<±0.5	<±0.5	±1.0	<±2	<±1	±0.05	±1	± 2 (±5 without internal reference)	±3 (±5 max)
Wavelength range (nm)	1510–1590	1510–1590	1460–1620	1500–1600	1500–1600	1500–1600	1528–1568	1528–1568	1529–1568	Up to 60	1525–1565 (1510–1590)	1525–1570 (1510–1595)
Sampling rate (Hz)	2	1000	1000	1	Up to 1000	1	5	2500	16	10–100	5 (Fast: ~5000)	
Number of optical channels	4	4 (up to 16)	1 or 4	1, 4, or 8	1, 4, or 8		1, 2, or 4	1, 2, or 4	4	18	1 or 4	
Maximum sensors	60–120	160 × 16		125–1000	125–1000		24–96	24–96	120			
Dynamic range (dB)	50	25	25 peak/40 FS	>50	>25	>50	38	38		>30		30
Internal wavelength reference	Yes	No	Yes	Yes	Yes	Yes	Yes	Yes	Yes	Yes	Option	

(*) Obtained over full temperature range; measurements accuracy carried out using calibrated instrument against an NIST traceable gas cell.

**Table 2:** Molecular gas cells as reference materials in the C and L bands for optical telecommunications.

Wavelength Standard Reference Material	Wavelength Range (nm)
^12^C_2_H_2_ [23]	1510–1540
^13^C_2_H_2_ [24]	1520–1550
H^12^C^14^N	1520–1555
H^13^C^14^N [25]	1530–1565
^12^C^16^O [26]	1560–1595
^13^C^16^O [26]	1595–1630

**Table 3:** Calculation of uncertainties of the first method experimental setup at $\lambda_{WM}\;\approx 1530\;\mathrm{nm}$.

Magnitude	Component of Uncertainty	Value (pm)	Type	Uncertainty Contribution (pm)
$\lambda_{WM}$	Wavelength meter (WM) calibration	9.4 × 10^−2^	B	4.7 × 10^−2^
	Linewidth of FBG	1.4 × 10^0^	B	3.9 × 10^−1^
	Repeatability of reference	4.0 × 10^−1^	B (*)	2.3 × 10^−1^
$\lambda_{FBGI}$	Optical resolution	8.0 × 10^−1^	B	2.3 × 10^−1^
	Display resolution	1.0 × 10^−2^	B	2.9 × 10^−3^
	On-off repeatability	2.0 × 10^−1^	B (*)	1.2 × 10^−1^
	Expanded uncertainty (U) (*k* = 2)			±1.1 × 10^0^

(*) Obtained by experimentally estimating the lower and upper bounds.

**Table 4:** Calculation of uncertainties of the second method experimental setup at $\lambda_{WM}\;\approx 1530\;\mathrm{nm}$ and for a period of time of 30 min, corresponding to *n* = 2245.

Magnitude	Component of Uncertainty	Value (pm)	Type	Uncertainty Contribution (pm)
$\lambda_{ref}$	Absorption line value	3.5 × 10^−1^	B	1.8 × 10^−1^
*λ_FBGI_*	Measurement stability	6.0 × 10^−4^	A	1.3 × 10^−5^
	Optical resolution	8.0 × 10^−1^	B	2.3 × 10^−1^
	Display resolution	1.0 × 10^−2^	B	2.9 × 10^−3^
	On-off repeatability	2.0 × 10^−1^	B (*)	1.2 × 10^−1^
	Expanded uncertainty (U) (*k* = 2)			±6.3 × 10^−1^

(*) Obtained by experimentally estimating the lower and upper bounds.

**Table 5:** Mean correction constants and expanded uncertainties (*k* = 2) for the four commercial systems calibrated.

Commercial System	Method 1			Method 2	
	*K_λ_* (pm)	*U* (*k* = 2) (pm)		*K_λ_* (pm)	*U* (*k* = 2) (pm)
sm125_unit 1	−0.9	±1.1		−0.23 (*)	±0.64 (*)
				−0.83 (**)	±0.63 (**)
sm125_unit 2				−0.08 (*)	±0.59 (*)
				−0.68 (**)	±0.61 (**)
FS42	−0.6	±1.1			
	
sm130	16.4	±1.1			
	

(*) 1515 nm–1550 nm; (**) 1560 nm–1570 nm.
